# A microscopic investigation on insulin uptake in human hepatocellular carcinoma-derived HepG2 cells

**DOI:** 10.1039/d5ra06143a

**Published:** 2025-11-05

**Authors:** Megren H. A. Fagihi, Yuchan Lee, Bridget Hogg, Massimiliano Garré, Sourav Bhattacharjee

**Affiliations:** a School of Medicine, University College Dublin Belfield Dublin 4 Ireland; b Conway Institute of Biomolecular and Biomedicine Research, University College Dublin Belfield Dublin 4 Ireland sourav.bhattacharjee@ucd.ie +353 1 716 6271; c Clinical Laboratory Sciences Department, College of Applied Medical Sciences, Najran University Najran 55461 Kingdom of Saudi Arabia; d School of Veterinary Medicine, University College Dublin Belfield Dublin 4 Ireland; e Super-Resolution Imaging Consortium, Department of Chemistry, Royal College of Surgeons in Ireland University of Medicine and Health Sciences Dublin D02 YN77 Ireland; f UCD One Health Centre, University College Dublin Belfield Dublin 4 Ireland; g UCD Earth Institute, University College Dublin Belfield Dublin 4 Ireland; h Institut für Funktionelle Anatomie, Charité – Universitätsmedizin Berlin, Campus Charité Mitte Philippstraße 11 10115 Berlin Germany

## Abstract

An *in vitro* study was conducted to investigate the cellular uptake and compartmentalization of internalized insulin in human hepatocellular carcinoma-derived HepG2 cells using confocal laser scanning microscopy. The cellular nuclei and actin filaments were stained with Hoechst (*λ*_ex_ = 405 nm, *λ*_em_ = 415–485 nm) and Alexa Phalloidin 647 (*λ*_ex_ = 649 nm, *λ*_em_ = 658–775 nm) dyes, respectively. Besides, recombinant human insulin, labeled with fluorescein isothiocyanate (*λ*_ex_ = 491 nm, *λ*_em_ = 502–600 nm), was used for the uptake studies (37 °C) at a concentration of 0.5 mg mL^−1^, with timepoints at 15 min and 30 min. The optimized tri-staining protocol proved adequate for confocal microscopy, while the 2D and 3D renditions revealed insulin uptake by HepG2 cells. The internalized (fluorescent) insulin was taken up into vesicles (receptor-mediated endocytosis) and spread throughout the cytoplasm, with little or no interaction with the actin network. Some of the internalized insulin accessed the cellular nuclei. Such nuclear interaction might explain insulin's role in influencing translation, cellular proliferation, and (overall) growth. The study developed an optimized tri-staining procedure that enabled in-depth visualization of HepG2 cells (actin network and nuclei) with internalized insulin, providing a better understanding of insulin's cellular uptake and nuclear interaction.

## Abstract 2

This study investigates the cellular uptake and intracellular fate of fluorophore-labeled human insulin in hepatocellular carcinoma-derived HepG2 cells using confocal microscopy.

## Graphical abstract

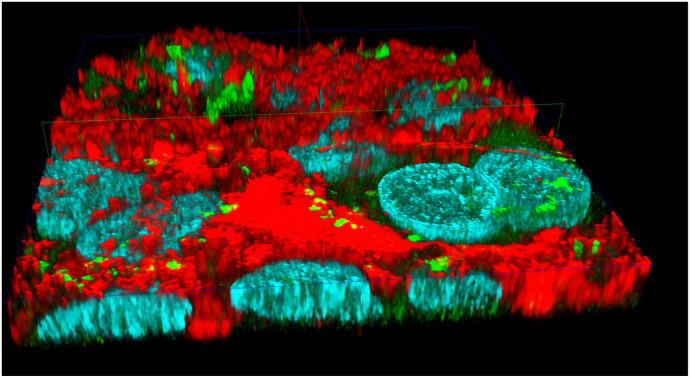

## 1. Introduction

Insulin, especially due to its critical role in regulating blood glucose levels (normal blood sugar levels: 70–100 mg dL^−1^ (3.9–5.6 mmol L^−1^) while fasting, and <140 mg dL^−1^ (7.8 mmol L^−1^) post-prandial), is the mainstay of therapeutic strategy in diabetes mellitus.^1,2^ As a peptide (molecular weight 5808 Da), it exerts a hypoglycemic impact by facilitating cellular uptake of glucose, thereby addressing hyperglycemia in diabetes.^3^ It is vital in treating Type I diabetes, where endogenous insulin production is compromised due to autoimmune destruction of the pancreatic β-cells responsible for insulin production, pre-processing, and storage.^4,5^ An inclusion in the World Health Organization's Model List of Essential Medicines further underscores its significance in current healthcare.^6^

**Fig. 1:**
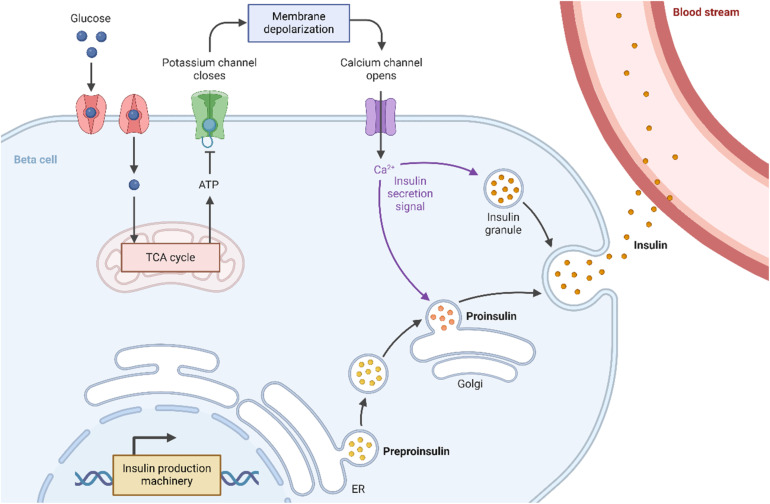
Scheme showing insulin's mechanism of action, driving blood glucose levels down. Insulin is produced as preproinsulin by the endoplasmic reticulum (ER) of the pancreatic β-cells, followed by processing in the Golgi apparatus to form proinsulin that is further matured into insulin-containing granules. These granules release insulin into the bloodstream, triggered by a Ca^2+^-dependent signaling cascade due to membrane depolarization resulting from potassium channel closure.

Insulin is released into the bloodstream by the pancreatic cells as a response to an elevated blood sugar level (Fig. 1). Once released, insulin interacts with target tissues—primarily the hepatic, muscular, and adipose tissues—to facilitate glucose uptake,^7^ mediated through insulin binding to the insulin receptors in the cell membrane affiliated with the tyrosine kinase superfamily.^8,9^ Upon insulin binding, the insulin receptors undergo autophosphorylation, resulting in the translocation of the glucose transporter type 2 (liver and pancreas)^10,11^ or type 4 (muscles and fat)^12,13^ to the cell membrane,^14^ which enhances glucose absorption from the blood and reduces blood sugar levels.

Another interesting and emerging topic of discussion is insulin's interaction with the cellular nuclei after cellular uptake,^15–17^ which, unfortunately, is often masked by insulin's major therapeutic role in lowering blood sugar levels in diabetes patients. It is known that post-uptake, a minute fraction of the internalized insulin is trafficked to the cellular nuclei, where insulin has been reported to influence various nuclear processes, including RNA synthesis,^18,19^ and the phosphorylation/dephosphorylation of nuclear proteins.^20,21^ Such insulin–nuclei interactions were reported to exert long-term effects on gene expression,^22^ cell proliferation,^23^ and metabolic processes,^24^ including carbohydrate metabolism^25^ and the cellular resilience against oxidative stress.^26^

The HepG2 is a popular immortalized cell line derived from human hepatocellular carcinoma, and serves as an *in vitro* model of polarized hepatocytes.^27^ They retain key morphological and functional attributes of the hepatic epithelial cells, including their morphology and the distinct apical and basolateral surfaces.^28^ Furthermore, they demonstrate responsiveness toward endocrine exposure, including growth hormone and insulin.^29^ Such attributes, coupled with its polarized morphology, make HepG2 cells an adequate *in vitro* model to investigate hepatic intracellular uptake,^30^ trafficking,^31^ and transport mechanisms in studies related to hepatic drug metabolism,^32^ various pathophysiology,^33^ drug design,^34^ and therapeutics (including targeting).^35^

In this study, we sought to integrate the advantages of HepG2 cells as an adequate hepatic model with advanced microscopy to image the cellular uptake and compartmentalization of insulin. Human insulin labeled with fluorescein isothiocyanate (FITC) was suitable for such investigations due to its photostability and adequate emission enabling intracellular imaging.^36^ To this end, this study aimed to develop a tri-staining protocol that, in conjunction with FITC-labeled insulin, also stained the cellular nuclei and actin filaments. Such simultaneous staining enabled a detailed investigation into how insulin is taken up by the HepG2 cells and its fate after uptake.

The aims and objectives of this study were: (i) to procure FITC-labeled (human) insulin; (ii) to stain the nuclei and actin filaments of the HepG2 cells with the Hoechst and Alexa Fluor Plus 647 Phalloidin dyes, respectively; (iii) to culture the HepG2 cells in chambered microslides, and expose them to FITC-insulin for various timepoints; (iv) to fix the exposed HepG2 cells and image them using confocal laser scanning microscopy (CLSM); (v) to understand the cellular uptake of FITC-insulin and identify its compartmentalization in HepG2 cells; (vi) to probe if the FITC-insulin interacts with the nuclei after internalization; (vi) to analyze the data and interpret the results from a therapeutics perspective.

The cumulative data provided valuable insights into the uptake of FITC-labeled insulin in HepG2 cells, with an understanding of its interactions with the nuclei and actin filaments. Moreover, the acquired knowledge can be utilized for facilitated cellular delivery of insulin.

## 2. Materials and methods

### 2.1. Chemicals and reagents

#### 2.1.1. Insulin

Human insulin, prepared by recombinant DNA technology and labeled with FITC (1 : 1 molecular substitution), was purchased from Sigma-Aldrich (Catalog No. I3661) and used as received without further purification.^7^ The powdered samples were stored in the dark at −20 °C.

#### 2.1.2. Cell culture ingredients

Paraformaldehyde (PFA) was procured commercially as a methanol-free 16% solution from Thermo Fisher Scientific (Catalog No. 11490570). The ProLong™ Diamond Antifade Mountant was purchased from Thermo Fisher Scientific (Catalog No. P36970).^37^

#### 2.1.3. Staining reagents

Alexa Fluor Plus 647 Phalloidin to stain cellular actin^38^ was purchased from Life Technologies Europe BV (Catalog No. A30107), while the Hoechst from Thermo Fisher Scientific (Catalog No. H21491).^39^

### 2.2. Cell culture and exposure to FITC-insulin

The HepG2 cells (resourced from The European Collection of Authenticated Cell Cultures (ECACC) with a Catalog No. 85011430) were maintained in a humidified incubator at 37 °C with 5% CO_2_ and were cultured in Dulbecco's Modified Eagle Medium (DMEM) supplemented with 10% fetal bovine serum (FBS) and 1% penicillin-streptomycin. For insulin exposure experiments, approximately 10 000 cells were seeded in Nunc Lab-Tek chamber microslides, and allowed to adhere and grow for 72 h. The cells were then treated with FITC-labeled human insulin (0.5 mg mL^−1^) for 15 min or 30 min at 37 °C. Following treatment, the medium was carefully removed, and the cells were washed thrice in phosphate-buffered saline (PBS). Only DMEM medium without insulin was used as the negative control.

Cellular fixation was performed by adding 200 μL of 4% PFA in PBS for 20 min, followed by three washes in PBS to remove the excess PFA. Subsequently, the cells were permeabilized using 0.1% Triton X-100 for 15 min, followed by additional washing (2×) with PBS. For actin staining, cells were first incubated with Alexa Phalloidin 647 for an hour, and then washed thrice in PBS. Subsequently, nuclear staining was done using Hoechst 33342 (5 μg mL^−1^) for 5 min, followed by two washes in PBS. The stained cells were mounted with ProLong™ Diamond Antifade Mountant, while the microslides were sealed and stored in the dark at 4 °C. The staining was performed at two time points (15 min, 30 min) in duplicate across two biological replicates. No staining was observed in any of the controls when the FITC-insulin was not present (control).

### 2.3. CLSM study

The CLSM was performed in a Leica Stellaris 8 Falcon System with a White Laser Source at room temperature (21 °C). Images were acquired with a Leica HC PL APO CS2 100×/1.40 oil immersion objective and with a zoom value of 2.5×. The Hoechst-stained cellular nuclei were imaged at *λ*_ex_ = 405 nm and *λ*_em_ = 415–485 nm, while the FITC-labeled insulin at *λ*_ex_ = 491 nm and *λ*_em_ = 502–600 nm, and the Alexa Phalloidin 647-stained actin filaments at *λ*_ex_ = 649 nm and *λ*_em_ = 658–775 nm. Digital 3D reconstructions of the cells, with stained intracellular actin, nuclei, and insulin, were generated from the *z*-stacks. No autofluorescence from the cells was detected under identical acquisition settings in the absence of all three stains.

**Fig. 2:**
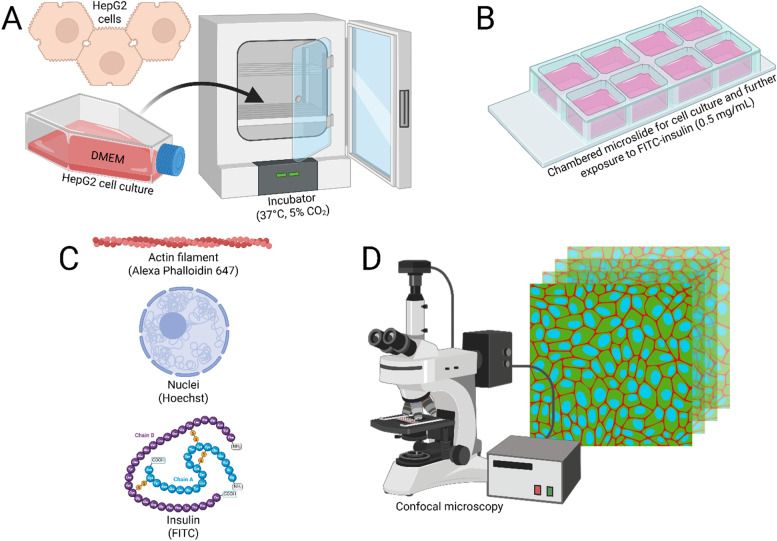
Flowchart showing the various steps of this study. (A) The HepG2 cells were grown in DMEM medium and maintained at 37 °C and 5% CO_2_. (B) The cells were then cultured in eight-well microslides with coverslip bottoms for exposure to FITC-insulin (0.5 mg mL^−1^) for *t* = 15 min and 30 min. (C) The stained components were the cellular nuclei, insulin, and actin, which were labeled with Hoechst, FITC, and Alexa Phalloidin 647, respectively. (D) CLSM was conducted for visualization of the nuclei (*λ*_ex_ = 405 nm, *λ*_em_ = 415–485 nm), FITC-insulin (*λ*_ex_ = 491 nm, *λ*_em_ = 502–600 nm), and the actin filaments (*λ*_ex_ = 649 nm, *λ*_em_ = 658–775 nm), with 3D rendition of the cells and internalized FITC-insulin.

A flowchart showing the major steps of the protocol followed in this study is shown in Fig. 2.

## 3. Results

**Fig. 3:**
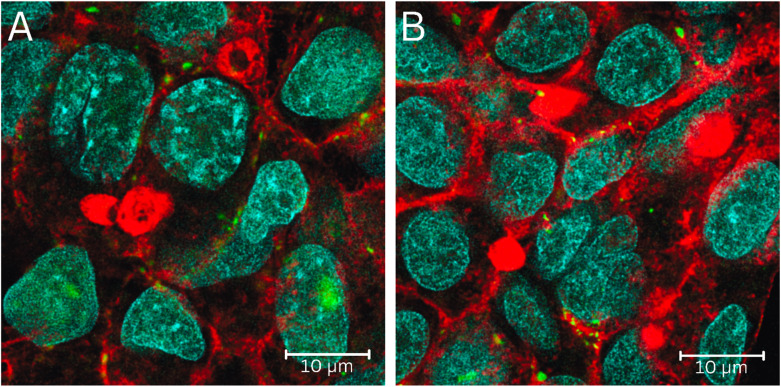
CLSM acquisition on the fixed HepG2 cells with internalized FITC-insulin after exposure to FITC-insulin (0.5 mg mL^−1^) for: (A) 15 min, and (B) 30 min. The cellular nuclei (blue), insulin (green), and actin (red) were labeled with Hoechst (*λ*_ex_ = 405 nm, *λ*_em_ = 415–485 nm), FITC (*λ*_ex_ = 491 nm, *λ*_em_ = 502–600 nm), and Alexa Phalloidin 647 (*λ*_ex_ = 649 nm, *λ*_em_ = 658–775 nm), respectively. A 10 μm scale bar is shown.

**Fig. 4:**
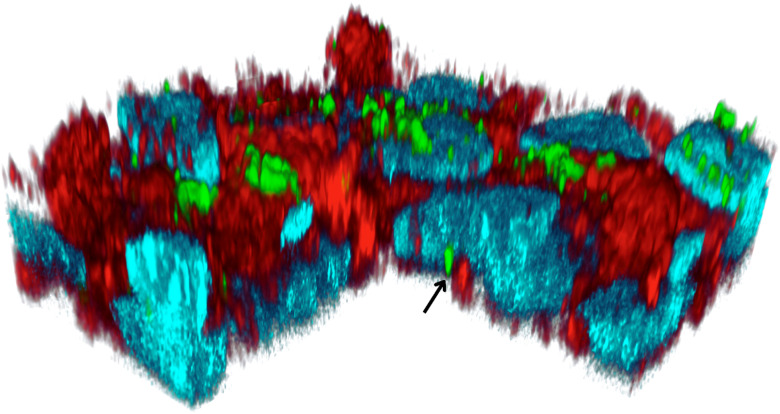
A 3D rendition of the HepG2 cells showing the intracellular distribution of the FITC-insulin after *t* = 30 min exposure. The cellular nuclei (blue), insulin (green), and actin (red) were labeled with Hoechst (*λ*_ex_ = 405 nm, *λ*_em_ = 415–485 nm), FITC (*λ*_ex_ = 491 nm, *λ*_em_ = 502–600 nm), and Alexa Phalloidin 647 (*λ*_ex_ = 649 nm, *λ*_em_ = 658–775 nm), respectively. The arrow points toward an insulin vesicle inside the nucleus.

**Fig. 5:**
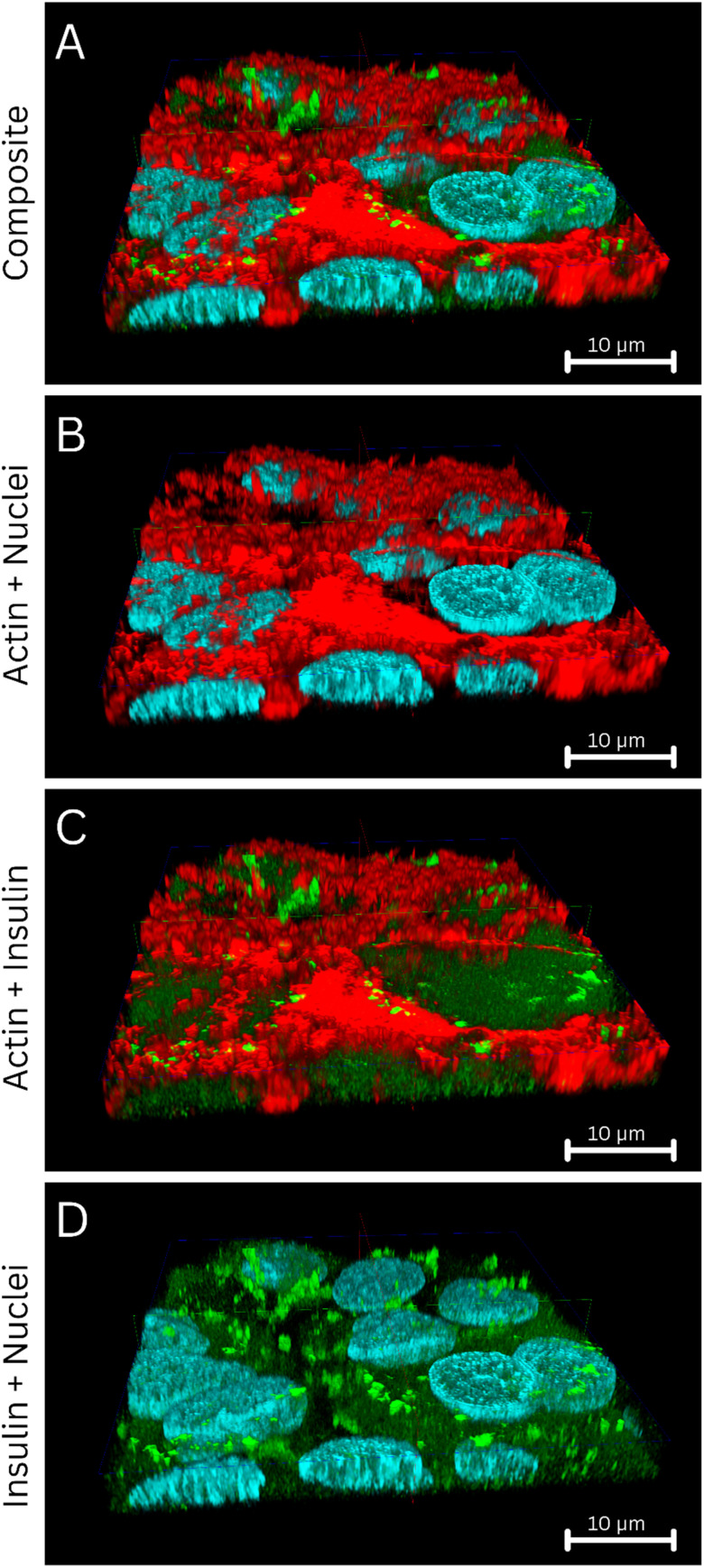
An orthogonal section of the 3D rendition of the HepG2 cells showing the intracellular distribution of the FITC-insulin. The cellular nuclei (blue), insulin (green), and actin (red) were labeled with Hoechst (*λ*_ex_ = 405 nm, *λ*_em_ = 415–485 nm), FITC (*λ*_ex_ = 491 nm, *λ*_em_ = 502–600 nm), and Alexa Phalloidin 647 (*λ*_ex_ = 649 nm, *λ*_em_ = 658–775 nm), respectively. (A) Composite representation of the tri-stained protocol followed in this study, where the nuclei, insulin, and actin filaments are visible. The frontal segment of the 3D view is sectioned orthogonally to demonstrate the interiors of the nuclei. (B) Only the nuclei (sectioned open) and actin are shown, demonstrating little or no interaction between them. (C) Only the actin and insulin are shown, demonstrating little or no interaction. (D) Only the insulin and nuclei (sectioned open) are shown, demonstrating interaction, while some insulin was identified inside the nuclei, indicating interaction. A 10 μm scale bar in the *x*–*z* plane is shown in each panel.

The tri-staining protocol (Hoechst-stained nuclei, FITC-labeled insulin, and Alexa Phalloidin 647-stained actin) worked adequately, and the simultaneous acquisition of stained nuclei, actin, or internalized insulin provided a 3D understanding of how the FITC insulin was taken up by the HepG2 cells, followed by its cellular compartmentalization (Fig. 3). In semi-quantitative terms, the HepG2 cells showed time-dependent uptake of FITC-insulin (30 min > 15 min). Some FITC-insulin was noted inside the nuclei (Fig. 4). A 3D rendering of cells with internalized FITC-insulin, created from *z*-stacks, revealed further details on the clustering of FITC-insulin and its nuclear uptake (Fig. 5 and S1–S3).

**Fig. 6:**
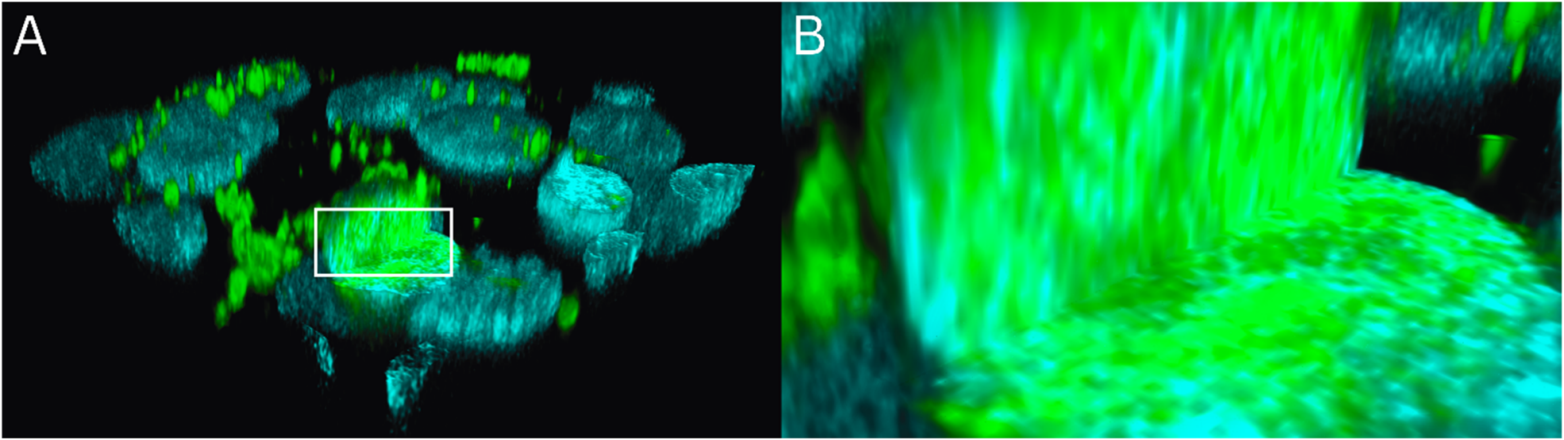
(A) An orthogonal section of the 3D rendition of the HepG2 cells showing the intracellular distribution of the FITC-insulin. The cellular nuclei (blue) and insulin (green) were labeled with Hoechst (*λ*_ex_ = 405 nm, *λ*_em_ = 415–485 nm) and FITC (*λ*_ex_ = 491 nm, *λ*_em_ = 502–600 nm), respectively. The sectioned nuclei clearly showed the presence of FITC-insulin inside them. (B) A zoomed view of the rectangular region of interest—marked in panel (A) as a white rectangle—is shown with a closer view of the nuclear interior. The FITC-insulin is interspersed with the nuclear genetic material.

The (stained) actin filaments did not show any interaction with the internalized FITC-insulin, and minimal/no colocalization between the emission from the actin filaments and FITC-insulin was noted (Fig. 5A–D). Upon further investigation, the FITC-insulin taken up by the cellular nuclei was observed to be distributed across the nuclear matrix rich in genetic material (Fig. 6A). A closer look further elucidated the pattern of how the FITC-insulin was interspersed within the nuclei (Fig. 6B).

**Fig. 7:**
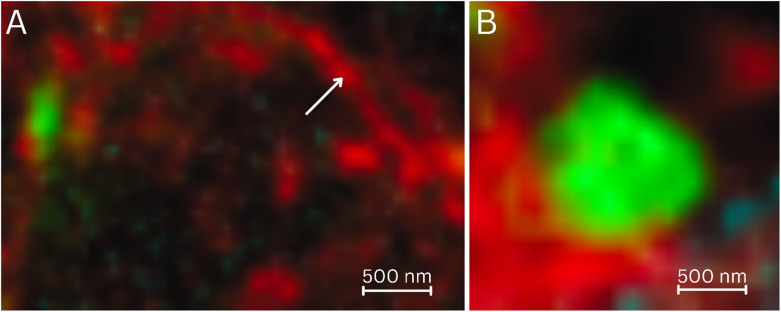
(A) The actin network stained with Alexa Phalloidin 647 (*λ*_ex_ = 649 nm, *λ*_em_ = 658–775 nm) and imaged by CLSM. An individual actin filament is marked with a white arrow. (B) An individual FITC-insulin (*λ*_ex_ = 491 nm, *λ*_em_ = 502–600 nm) vesicle of ∼1 μm size taken up by the HepG2 cells through receptor mediation (*t* = 30 min). A 500 nm scale bar is provided in each panel.

Individual actin filaments were also identified in CLSM (Fig. 7A). The internalized FITC-insulin in HepG2 cells was present in vesicles (Fig. 7B), indicating a receptor-mediated cellular uptake.

## 4. Discussion

The tri-staining protocol, followed by microscopic evaluation, enabled both 2D and 3D visualization of HepG2 cells with internalized FITC-insulin. Such a protocol needed extensive optimization, although the results were satisfactory and provided a better understanding of how insulin was taken up by these cells and of its post-internalization fate. The HepG2 cells worked well as an *in vitro* model for the investigation, as they are known to express insulin receptors adequately.^40^ In a way, the method of such tri-staining can be extended beyond the realms of insulin only, and with the availability of a wide array of fluorophores, the scope of such labeling-based confocal microscopy, or any advanced microscopy *per se*, is quite broad and keeps evolving at a rapid rate.

A relatively higher concentration of insulin (0.5 mg mL^−1^ ≈ 14.4 IU mL^−1^) compared to normal human insulin levels in blood (5–15 μIU mL^−1^ in fasting state)^41^ was administered to ensure that excess insulin was available to the cells, and the cellular uptake of insulin was dose independent. Under the studied conditions, it is difficult to achieve a quantifiable dose response while working on a monolayer of HepG2 cells grown on a glass coverslip. However, techniques like flow cytometry may be used in the future to determine if such a dose response exists.^42,43^ The aim of this study, however, was to determine whether FITC-labeled insulin uptake was observable in these cells with its compartmentalization, which was achieved microscopically.

The human insulin molecule (*M*_W_ ∼ 5808 Da) is much larger than the FITC (*M*_W_ ∼ 390 Da) molecule, and the degree of substitution (labeling efficiency) in the used FITC-insulin was 1 : 1; thus, (roughly) one molecule of FITC was conjugated to one molecule of insulin. Taken together, FITC is too small a molecule and present in insufficient amounts to exert any significant biochemical influence on the insulin molecule or to alter its molecular behavior in a physiologically relevant milieu, including its binding to insulin receptors. Previous publications from our lab also did not detect any alteration in the physicochemical properties of FITC-insulin compared to unconjugated insulin.^7^ Within the scope of this study, based on fluorescence microscopy, using unlabeled insulin was also not feasible as a possible control and was therefore omitted.

Additionally, when conjugated to insulin, FITC was an excellent fluorophore for biophotonic evaluation. It was photostable, and, at least under the protocols followed in this study, no photobleaching or spectral overlap with the other two dyes (Hoechst and Alexa Phalloidin 647) was observed. Earlier work from our lab on the same FITC-insulin was able to capitalize on the pH-dependent fluorescence lifetime (*τ*) variation of FITC to develop a molecular pH meter using fluorescence lifetime imaging microscopy, while the FITC-insulin conjugation was robust with no observed dissociation of the conjugated FITC from the labeled insulin molecules.^7,44^

The internalized insulin demonstrated a uniform distribution throughout the cytoplasm, with little or no interaction with the intracellular actin network. The microscopy data identified spherical vesicular structures containing FITC-insulin within the cells, indicating receptor-mediated uptake.^45^ Interestingly, insulin is known to form agglomerates when subjected to fluctuations in its physicochemical microenvironment, including (but not limited to) application of heat, agitation, and acidity.^3^ Previous data from our lab have established that, depending on circumstances, these agglomerates can be as large as a few μm.^7^ Such large agglomerates were incompatible with binding to the insulin receptors located at the cell membrane. However, ongoing studies in our lab (data not shown) have also demonstrated that under ambient physiological conditions and pH, such agglomerates disintegrated over time, especially in media rich in ions, such as the DMEM cell culture medium used in this study. Thus, the cells might have interacted with insulin in either a molecular state or as small agglomerates that engaged the insulin receptors of HepG2 cells, enabling vesicular cellular uptake.

The internalized FITC-insulin did not show any interaction with the intracellular actin. The available literature did not report any direct insulin–actin binding, which was consistent with our data. However, it is worth noting that, despite no direct binding, insulin is known to influence actin remodeling, or the recruitment of proteins in actin filaments.^46^ Conversely, the actin filaments impact the transport of glucose transporters^47^ and insulin secretion.^48^

The internalized insulin permeated the nuclear membrane and accessed the nuclear interior. Such nuclear interactions of insulin are a rapidly evolving field of research that sheds light on insulin's bioactivity beyond merely lowering blood sugar levels. Insulin's role in the human body is multifaceted, and it is part of a much larger family of growth factors, for example, the insulin-like growth factors.^49–51^ Previous studies have shown that nuclear transport of insulin is mediated by insulin receptors on nuclear membranes, which are overexpressed following insulin exposure.^52,53^ Such insulin uptake by the cellular nuclei influences gene expression, leading to cellular proliferation and growth, as well as metabolic regulation.^15^ Insulin is known to modulate the FOXO (stress response, metabolism, and cell division),^54,55^ SREBP (synthesis of cholesterol, fatty acids, and other lipids),^56^ and Sp1 (growth, differentiation, and survival)^57^ transcription factors by influencing their DNA-binding capabilities. Similarly, insulin enhances the transcription of genes encoding enzymes involved in glycolysis^58^ and lipogenesis,^59^ while inhibiting genes involved in gluconeogenesis.^60^ Our results aligned well with the reported data and reinforced the current concepts of insulin–nucleus interaction.

## 5. Conclusion

A confocal microscopy study was conducted on (fixed) HepG2 cells to investigate the cellular uptake and post-uptake fate of FITC-insulin. The followed tri-staining protocol enabled simultaneous labeling of the cellular nuclei with Hoechst and the actin network with the Alexa Phalloidin 647 dye, and traced the internalized insulin within a 3D intracellular environment. The results confirmed cellular uptake of FITC-insulin in vesicles, indicative of receptor-mediated uptake. These vesicles were distributed throughout the cytoplasm, with no recognizable interactions with the actin network. However, some of the internalized insulin penetrated the nuclear membrane, indicating nuclear interaction with implications for gene expression and cellular proliferation. The cumulative data provided a better understanding of how insulin was taken up by the HepG2 cells, with relevance for cellular delivery.

## Author contributions

M. H. A. Fagihi (investigation, formal analysis, visualization, writing – original draft), Y. Lee (investigation, formal analysis, visualization, writing – review and editing), B. Hogg (investigation, supervision, formal analysis, visualization, supervision, writing – review and editing), M. Garré (investigation, formal analysis, visualization, writing – review and editing), S. Bhattacharjee (investigation, formal analysis, visualization, resources, writing – review and editing).

## Conflicts of interest

The authors have no relevant affiliations or financial involvement with any organization or entity with a financial interest in or financial conflict with the subject matter or materials discussed in the manuscript. This includes employment, consultancies, honoraria, stock ownership or options, expert testimony, grants or patents received or pending, or royalties. No writing assistance was utilized in the production of this manuscript.

## Supplementary Material

RA-015-D5RA06143A-s001

RA-015-D5RA06143A-s002

RA-015-D5RA06143A-s003

RA-015-D5RA06143A-s004

RA-015-D5RA06143A-s005

## Data Availability

Further data may be obtained from the corresponding author upon reasonable request. The data supporting this article have been included as part of the supplementary information (SI). Supplementary information is available. See DOI: https://doi.org/10.1039/d5ra06143a.
