# Supplementary material for: A microscopic investigation on insulin uptake in human hepatocellular carcinoma-derived HepG2 cells
Source: RSC Adv. 2025 Nov 5;15(50):42605–13. doi: 10.1039/d5ra06143a (PMC12588072; doi:10.1039/d5ra06143a)
Supplement: RA-015-D5RA06143A-s005 [file RA-015-D5RA06143A-s005.pdf]

Supplementary Information (SI) for RSC Advances.
This journal is © The Royal Society of Chemistry 2025

SUPPORTING INFORMATION

A microscopic investigation on insulin uptake in the human hepatocellular carcinoma-derived HepG2 cells

Megren H. A. Fagihi,[1,2,3,§] Yuchan Lee,[2,4,§] Bridget Hogg,[2,4]
Massimiliano Garré,[5] Sourav Bhattacharjee[2,4,6,7,8,*]

[1] School of Medicine, University College Dublin, Belfield, Dublin 4, Ireland

[2] Conway Institute of Biomolecular and Biomedicine Research, University College Dublin, Belfield, Dublin 4, Ireland

[3] Clinical Laboratory Sciences Department, College of Applied Medical Sciences, Najran University, Najran 55461, Kingdom of Saudi Arabia

[4] School of Veterinary Medicine, University College Dublin, Belfield, Dublin 4, Ireland

[5] Super-Resolution Imaging Consortium, Department of Chemistry, Royal College of Surgeons in Ireland University of Medicine and Health Sciences, Dublin D02 YN77, Ireland

[6] UCD One Health Centre, University College Dublin, Belfield, Dublin 4, Ireland

[7] UCD Earth Institute, University College Dublin, Belfield, Dublin 4, Ireland

[8] Institut für Funktionelle Anatomie, Charité – Universitätsmedizin Berlin, Campus Charité Mitte, Philippstraße 11, 10115 Berlin, Germany

[§]Equal contribution

*Corresponding author

Email: sourav.bhattacharjee@ucd.ie

T.: +353 1 716 6271

S1. A step-by-step visualization from a 3D rendition of a Hoechst-stained HepG2 cell nucleus showing its internal texture along with intrusion by FITC-insulin. The Alexa Phalloidin 647-stained actin filaments did not demonstrate any interaction with the nucleus.

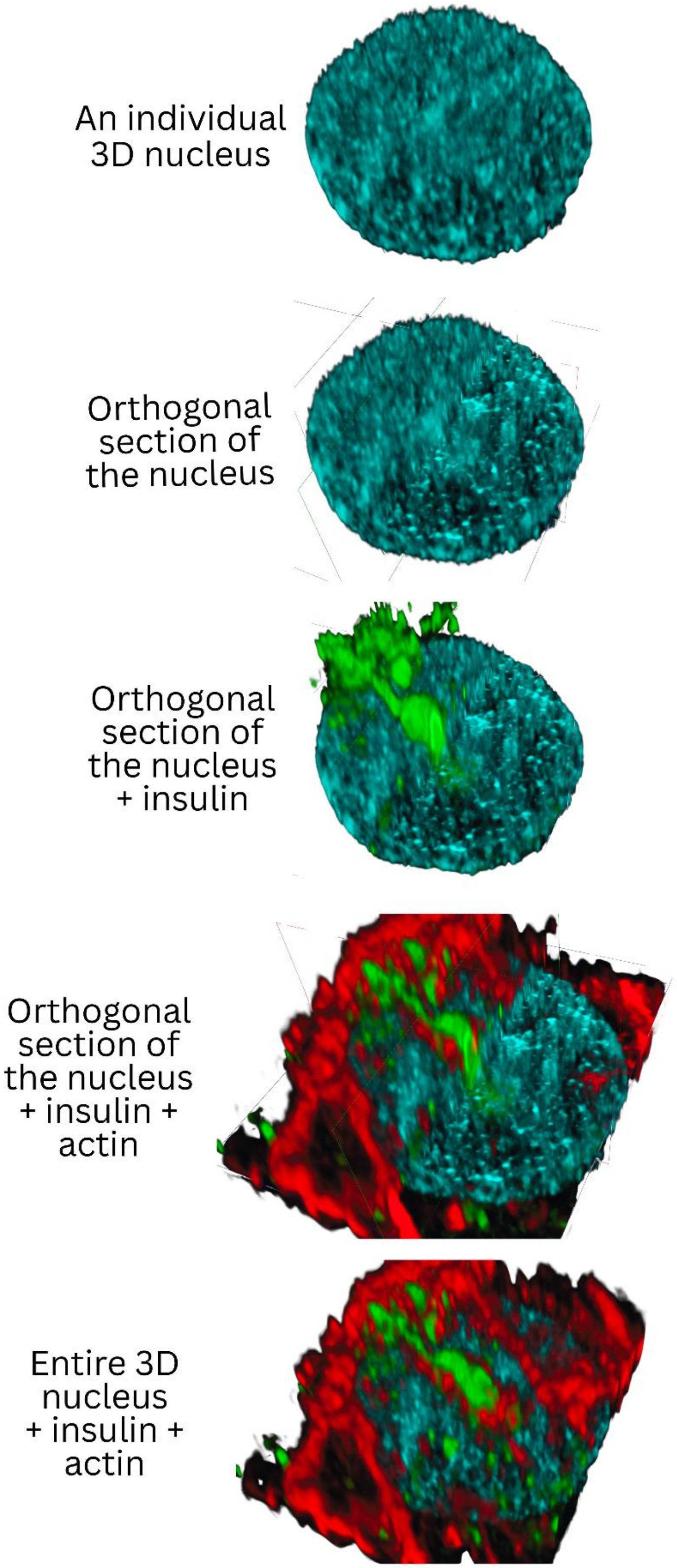

S2. Video file (marked as S1_15 min) showing the 3D rendition of the HepG2 cells derived from the CLSM data after t = 15 min exposure to FITC-insulin with the nuclei, internalized FITC-insulin, and the actin filaments stained with Hoechst (λ_{ex}=405 nm, λ_{em}=415–485 nm), FITC (λ_{ex}=491 nm, λ_{em}=502–600 nm), and Alexa Phalloidin 647 (λ_{ex}=649 nm, λ_{em}=658–775 nm), respectively.

S3. Multiple video files (marked as S2_30 min_1/2/3) showing the 3D rendition of the HepG2 cells derived from the CLSM data after t = 30 min exposure to FITC-insulin with the nuclei, internalized FITC-insulin, and the actin filaments stained with Hoechst (λ_{ex}=405 nm, λ_{em}=415–485 nm), FITC (λ_{ex}=491 nm, λ_{em}=502–600 nm), and Alexa Phalloidin 647 (λ_{ex}=649 nm, λ_{em}=658–775 nm), respectively.
